# Calbindin regulates Kv4.1 trafficking and excitability in dentate granule cells via CaMKII-dependent phosphorylation

**DOI:** 10.1038/s12276-021-00645-4

**Published:** 2021-07-07

**Authors:** Kyung-Ran Kim, Hyeon-Ju Jeong, Yoonsub Kim, Seung Yeon Lee, Yujin Kim, Hyun-Ji Kim, Suk-Ho Lee, Hana Cho, Jong-Sun Kang, Won-Kyung Ho

**Affiliations:** 1 Department of Physiology, Seoul National University College of Medicine, Seoul, Korea; 2 Department of Molecular Cell Biology, Sungkyunkwan University School of Medicine, Suwon, Korea; 3 Department of Brain and Cognitive Science, Seoul National University College of Natural Science, Seoul, Korea; 4 Department of Physiology, Sungkyunkwan University School of Medicine, Suwon, Korea; 5 Neuroscience Research Institute, Seoul National University College of Medicine, Seoul, Korea; 6 Present Address: Institute of BioInnovation Research, Kolon Life Science Inc, 110 Magokdong-ro, Gangseo-gu, Seoul, 07793 Korea

**Keywords:** Cellular neuroscience, Intrinsic excitability

## Abstract

Calbindin, a major Ca^2+^ buffer in dentate granule cells (GCs), plays a critical role in shaping Ca^2+^ signals, yet how it regulates neuronal function remains largely unknown. Here, we found that calbindin knockout (CBKO) mice exhibited dentate GC hyperexcitability and impaired pattern separation, which co-occurred with reduced K^+^ current due to downregulated surface expression of Kv4.1. Relatedly, manipulation of calbindin expression in HT22 cells led to changes in CaMKII activation and the level of surface localization of Kv4.1 through phosphorylation at serine 555, confirming the mechanism underlying neuronal hyperexcitability in CBKO mice. We also discovered that Ca^2+^ buffering capacity was significantly reduced in the GCs of Tg2576 mice to the level of CBKO GCs, and this reduction was restored to normal levels by antioxidants, suggesting that calbindin is a target of oxidative stress. Our data suggest that the regulation of CaMKII signaling by Ca^2+^ buffering is crucial for neuronal excitability regulation.

## Neuroscience: an important buffer for memory-making

Regulation of cytosolic calcium in neurons is crucial for normal brain functions. Neuronal signaling is governed in part by dynamiic changes in calcium concentrations, and calbindin acts as a ‘buffer’ that binds calcium ions to shape the spatiotemporal extent of changes in free calcium concentrations. Prior studies have noted reduced calbindin levels in certain neurological disorders, and researchers led by Won-Kyung Ho at Seoul National University, and Jong-Sun Kang at Sungkyunkwan University, South Korea, have uncovered a potential mechanism underlying these observations. Loss of calbindin in mouse models resulted in excessive firing by certain cells in a brain structure called the dentate gyrus. This structure is important to cognition, and the mice showed impaired performance in discriminating similar contexts, so-called “pattern separation”, highlighting the critical importance of calcium homeostasis in the brain.

## Introduction

Ca^2+^ signaling is involved in every aspect of neuronal function, from normal physiology, such as synaptic transmission and memory formation, to the pathogenesis underlying various neurological and psychiatric diseases. Cytosolic Ca^2+^ buffers are essential components for the maintenance of Ca^2+^ homeostasis and the shaping of Ca^2+^ signals^1^. Calbindin-D_28k_ (CB) is a major Ca^2+^ buffer of mature granule cells (GCs) in the dentate gyrus (DG) of the hippocampus^2^, where half of the Ca^2+^ buffering is attributable to CB^3^. CB is typically a mobile and fast-acting Ca^2+^ buffer that helps to shape the spatiotemporal extent of Ca^2+^ signals^4^. Increased CB levels in response to nerve growth factor^5^ and a lack of CB in the degenerated substantia nigra^6^ suggest that CB plays a protective role against excitotoxicity. Interestingly, the expression of CB in the DG is markedly reduced in various pathological conditions that are accompanied by cognitive dysfunction, including Alzheimer’s disease (AD)^7–9^ and schizophrenia/bipolar disorder^10–12^. Downregulation of CB in hippocampal excitatory neurons by early life stress is implicated in increased susceptibility to stress-induced memory deficits^13^. However, the current mechanistic understanding of the pathophysiological roles of CB reduction is poor.

Ca^2+^/calmodulin (CaM)-dependent protein kinase II (CaMKII) is a multifunctional serine/threonine protein kinase that is highly concentrated in the brain, and activity-dependent activation of CaMKII plays a central role in synaptic plasticity^14^ and neuronal excitability^15,16^. Abnormal CaMKII activity has been implicated in various neuronal diseases, including schizophrenia^11^, intellectual disability^17^, and AD^18^, highlighting the importance of CaMKII in neuronal function. Calcineurin, a Ca^2+^-dependent protein phosphatase, plays a critical role in the activity-dependent alteration of synaptic transmission^19^. Intriguingly, Ca^2+^ buffers affect Ca^2+^-dependent protein kinase and phosphatase activity, thereby regulating neuronal function. Overexpression of CB was found to promote neuronal differentiation, which was concurrent with CaMKII activation and inhibited by a CaMKII inhibitor^20^, suggesting a link between CaMKII and CB in hippocampal progenitor cells. It is of great interest whether the interplay between CB and CaMKII plays a role in mature neurons.

The DG of the hippocampus has long been postulated to mediate pattern separation by transforming similar inputs into distinct neural representations^21–23^. The sparse activity of GCs has been regarded as essential for the computational function that makes pattern separation possible^22–24^. We have recently discovered that Kv4.1 is a key ion channel for the low-frequency firing of GCs, and mice with Kv4.1 depletion in the DG show impaired pattern separation^25^, highlighting the role of Kv4.1 in DG functions. The regulatory mechanisms of Kv4.1 are an intriguing topic worthy of further investigation.

In this study, we discovered that the GCs in CB knockout (CBKO) mice show increased neuronal excitability along with reduced Kv4.1 activity and CaMKII activation. CB deficiency results in impaired CaMKII activation, which in turn reduces the surface localization of Kv4.1. CaMKII interacts with and phosphorylates the Kv4.1 protein at serine 555 (S555), which regulates its targeting to the membrane and its channel activity. Furthermore, CBKO mice showed impaired pattern separation. Our data collectively suggest that CB reduction predisposes patients to cognitive dysfunction at least partially by disrupting the CaMKII-dependent regulation of Kv4.1.

## Materials and methods

### Preparation of brain slices

Brain slices were prepared from calbindin-D_28k_ knockout (CBKO) mice (on a C57BL6/J genetic background) and control C57BL6/J mice aged 1–2 months old. CBKO mice were kindly provided by Dr. Schwaller (Univ. of Fribourg, Switzerland). The average ages of CBKO and control C57BL6/J mice used in the present study were 5.6 (*n* = 36) and 5.7 weeks (*n* = 10), respectively. Experiments for dentate GCs were mostly conducted using mice at postnatal week (PW) 4 to PW 7, while experiments for CA1-PCs were conducted using mice at PW 3 to PW 4. Mice were killed by decapitation after being anesthetized with isoflurane, and the whole brain was immediately removed from the skull and chilled in artificial cerebrospinal fluid (aCSF) at 4 °C. Transverse hippocampal slices (350-μm thick) were prepared using a vibratome (VT1200S, Leica, Germany). Slices were incubated at 35 °C for 30 min and subsequently maintained at 32 °C until in situ slice patch-clamp recordings and fluorescence microscopy. All experimental procedures were conducted in accordance with the guidelines of the University Committee on Animal Resource at Seoul National University (Approval No. SNU-090115-7).

### Electrophysiological analysis of excitability and K^+^ currents

Hippocampal GCs of the DG were visualized using an upright microscope equipped with differential interference contrast (DIC) optics (BX51WI, Olympus, Japan). Electrophysiological recordings were made using the whole-cell clamp technique with an EPC-8 amplifier (HEKA, Lambrecht, Germany). The experiments were performed at 32 ± 1 °C. After break-in, we waited 5 min for the neurons to stabilize. The perfusion rate of the bath solution and the volume of the recording chamber for slices were 2.2 mL/min and 1.2 mL, respectively. Patch pipettes with a tip resistance of 3–4 MΩ were used. The series resistance (R_s_) after the establishment of the whole-cell configuration was between 10 and 15 MΩ. The pipette solution contained the following (in mM): 143 K-gluconate, 7 KCl, 15 HEPES, 4 Mg–ATP, 0.3 Na–GTP, 4 Na-ascorbate, and 0.1 EGTA/or 10 BAPTA, with the pH adjusted to 7.3 with KOH. For the antibody blocking experiments, patch pipettes were dipped into an internal solution containing anti-Kv4.1 antibodies at a concentration of 0.3 µg/mL and were then back-filled with that solution. The bath solution (or aCSF) for the control experiments contained the following (in mM): 125 NaCl, 25 NaHCO_3_, 2.5 KCl, 1.25 NaH_2_PO_4_, 2 CaCl_2_, 1 MgCl_2_, 20 glucose, 1.2 pyruvate, and 0.4 Na-ascorbate, pH 7.4 when saturated with carbogen (95% O_2_ and 5% CO_2_). In all bath solutions, 20 μM bicuculline and 10 μM CNQX were included to block synaptic inputs. In voltage-clamp experiments to record K^+^ currents, we added TTX (0.5 µM), CdCl_2_ (300 µM), and NiCl_2_ (500 µM) to block Na^+^ and Ca^2+^ channels, and membrane potentials were depolarized to a maximum of 30 mV for 1 s in 10 mV increments from the holding potential of −70 mV. In current-clamp experiments to analyze neuronal excitability, the following parameters were measured: (1) the resting membrane potential (RMP), (2) the input resistance (R_in_, membrane potential changes (V) for the given hyperpolarizing current (−35 pA, 600 ms) input), (3) the F–I curve (firing frequencies (F) against the amplitude of injected currents (I), for DG; 100–600 pA, 100 pA increment, 1 s duration, for CA1 pyramidal cells (CA1-PCs); 50– 250 pA, 50 pA increment, 1 s duration), and (4) the AP onset time at 300 pA (the delay from the start of 300 pA injection to the beginning of the upstroke phase of the 1st evoked AP). All chemicals were obtained from Sigma (St. Louis, MO, USA), except for CNQX, bicuculline, and TTX, which were obtained from Abcam Biochemicals (Cambridge, UK).

### Cytosolic Ca^2+^ measurement and estimation of calcium-binding ratios

Cytosolic [Ca^2+^] was measured from fluorescence images of hippocampal GCs in slices loaded with fura-2 (pentapotassium salt). The slices were illuminated using a polychromatic light source (xenon lamp-based, Polychrome, Martinsried, Germany), which was coupled to the epi-illumination port of an upright microscope (BX51, Olympus, Japan) via a quartz light guide and a UV condenser, and images were captured with an air-cooled slow-scan CCD camera (SensiCam, PCO, Kelheim, Germany). The monochromator and the CCD camera were controlled by a PC and ITC18, running a custom-made software program written in Microsoft Visual C^++^ (version 6.0). Details of the calibration method have been described previously^26^.

To estimate the endogenous Ca^2+^ binding ratio (κ_E_), we applied short depolarizing pulses (from −70 to 0 mV, 40 ms in duration) every 20–60 s to evoke [Ca^2+^] transients in the mature dentate GCs. Normally, κ_E_ is estimated according to the single compartment and linear approximation model^27,28^. When two different Ca^2+^ buffers, a Ca^2+^ indicator dye (B) and an endogenous Ca^2+^ buffer (E), exist in the compartment, the increments of total and free calcium have the following relationship:

1 $${\Delta}[{\mathrm{Ca}}^{2 + }]_{\mathrm{T}} = {\Delta}[{\mathrm{Ca}}^{2 + }]_{\mathrm{i}}\cdot (1 + \kappa _{\mathrm{B}} + \kappa _{\mathrm{E}}),$$

where κ_B_ and κ_E_ are the calcium-binding ratios of B and S, respectively. Ca^2+^ transients (Δ[Ca^2+^](t), CaTs) following short pulses of Ca^2+^ influx can be described by the following equations:

2 $${\Delta}[{\mathrm{Ca}}^{2 + }]({\mathrm{t}}) = {\it{A}}{\mathrm{exp}}( - {\mathrm{t}}/\tau ),$$

3 $${\it{A}} = {\Delta}[{\mathrm{Ca}}^{2 + }]_{\mathrm{T}}/(1 + \kappa _{\mathrm{B}} + \kappa _{\mathrm{E}}),$$

4 $$\tau = (1 + \kappa _{\mathrm{B}} + \kappa _{\mathrm{E}})/\gamma ,$$

where *A* is the initial amplitude, d[Ca^2+^]_T_ is the total intracellular Ca^2+^ increase evoked by the influx, γ is the Ca^2+^ extrusion rate, and κ_B_ is the Ca^2+^ binding ratio of the Ca^2+^ indicator dye (fura-2). The calcium-binding ratio of a buffer X is defined by

5 $$k_{\mathrm{X}} = \partial [{\mathrm{CaX}}]/\partial [{\mathrm{Ca}}^{2 + }] = {\mathrm{K}}_{\mathrm{d}}\cdot {\mathrm{X}}_{\mathrm{T}}/([{\mathrm{Ca}}^{2 + }] + {\mathrm{K}}_{\mathrm{d}})^2,$$

where X_T_ and K_d_ are the total concentration of X and the dissociation constant of X for Ca^2+^, respectively^28^. When κ_X_ is not constant over the dynamic range of [Ca^2+^] in a Ca^2+^ transient, the incremental Ca^2+^-binding ratio, κ‘_X_, should be used. This ratio is defined as

6 $${\mathrm{k}}^\prime _{\mathrm{X}} = {\mathrm{{\Delta}}}[{\mathrm{CaX}}]/{\mathrm{{\Delta}}}[{\mathrm{Ca}}^{2 + }] = {\mathrm{K}}_{\mathrm{d}}\cdot {\mathrm{X}}_{\mathrm{T}}/[([{\mathrm{Ca}}^{2 + }]_{{\mathrm{i}},{\mathrm{rest}}} + {\mathrm{K}}_{\mathrm{d}}) \cdot ([{\mathrm{Ca}}^{2 + }]_{{\mathrm{i}},{\mathrm{peak}}} + {\mathrm{K}}_{\mathrm{d}})],$$

where [Ca^2+^]_i,rest_ and [Ca^2+^]_i,peak_ represent the [Ca^2+^]_i_ value before and at the peak perturbation, [X]_T_ is the total concentration of Ca^2+^ buffer X, and K_d_ is the Ca^2+^ dissociation constant of X^28^. According to Eqs. (3) and (4), plotting the *A* and τ values measured at different levels of κ_B_ provides two estimates of κ_E_: one is obtained from the *x*-intercept of the straight line fitted to the plot of τ vs. κ_B_, and the other is obtained from a line fitted to the plot of *A*^*−*1^ vs. κ_B_. These two estimates will be referred to as κ_E._

### Cell culture and constructs

HT22 (Sigma-Aldrich #SCC129) and HEK293T (ATCC CRL-3216) cells were cultured, as previously described^29,30^. HT22 and HEK293T cells were cultured in DMEM containing 10% fetal bovine serum (FBS, Gibco) and transiently transfected with a combination of DNA plasmids by using Lipofectamine 3000 (Invitrogen). To examine CB knockdown effects, HT22 cells were infected with a lentivirus carrying the control scrambled-shRNA (5′-CCGGCAACAAGATGAAGAGCACCAACTCGAGTTGGTGCTCTTCATCTTGTTGTTTTTG-3′) or CB-specific shRNA (5′-CCGGGATTGGAGCTATCACCGGAAACTCGAGTTTCCGGTGATAGCTCCAATCTTTTTG-3′) with polybrene (Sigma) for 2 days. To induce differentiation, cells at ~70–80% confluence were switched to Neurobasal medium (Gibco) containing N2, B27, GlutaMAX, 5 ng/mL BDNF, and 50 ng/mL NGF (Thermo Fisher). The constructs used in this study were as follows: pCI-neo, pCI-neo-CB, Kv4.1-GFP (Origene, MG220056) and GFP-C1-CaMKIIα (Addgene, 21226). To examine the role of Kv4.1 phosphorylation, a serine-alanine mutation was introduced at serine residue 265, 555, or 568 of Kv4.1 by using a mutagenesis kit (Stratagene).

The Kv4.1 currents from HEK293T cells were measured with the whole-cell patch-clamp technique. A voltage clamp was performed by using an EPC-10 amplifier (HEKA Instrument, Germany), and a 10 kHz filter was applied. The patch pipettes (World Precision Instruments, Inc., USA) were made with a Narishige puller (PP-830, Narishige Co, Ltd., Japan). The patch pipettes used in this study had a resistance of 2–3 MΩ when filled with the pipette solutions listed in the next sections. All recordings were carried out at room temperature. The normal external solution for HEK293T cell recording was as follows (in mM): 143 NaCl, 5.4 KCl, 5 HEPES, 0.5 NaH_2_PO_4_, 11.1 glucose, 0.5 MgCl_2_, 1.8 CaCl_2_, pH 7.4 after adjustment with NaOH. The pipette solution was as follows (in mM): 135 K-aspartate, 2 MgCl_2_, 3 EGTA, 1 CaCl_2_, 4 Mg–ATP, 0.1 Na–GTP, 10 HEPES, pH 7.4 after adjustment with KOH. Currents were analyzed and fitted using Patchmaster (HEKA Instrument) and Origin 6.1 (Originlab Corp., USA) software. All values are given as the mean ± SEM. Current–voltage (I/V) relations were obtained by plotting the outward current at 1 s into the test pulse as a function of the test potential. Current densities (pA/pF) were obtained after normalization to cell-surface area as calculated by Patchmaster.

### Protein analysis and surface biotinylation

Western blot analysis was performed as previously described^25,31^. Briefly, cells were lysed in RIPA lysis buffer (iNtRON) containing a complete protease inhibitor cocktail (Roche), followed by SDS-PAGE and incubation with primary and secondary antibodies. Primary antibodies against calbindin, p-CaMKIIα, CaMKIIα (Cell Signaling), Kv4.1, Kv4.2 (Alomone Lab), GFP (Abcam), and β-tubulin (Sigma) were used.

Immunoprecipitation was performed as previously described^32^. Briefly, precleared cell extracts were incubated with anti-phosphoserine antibodies (Santa Cruz) overnight at 4 °C, followed by incubation with protein G-agarose beads (Roche) for 1 h. Subsequently, the beads were washed three times with cell extraction buffer, and the precipitates were subjected to western blotting.

Surface biotinylation analysis was carried out as previously described^33^. In brief, the surface proteins of HT22 cells or mouse dentate gyrus were biotinylated by exposure to 1 mg/mL NHS-LC-biotin (Thermo) for 30–60 min at 4 °C. After a quenching reaction with 100 mM glycine, cells or dentate gyrus were lysed and sonicated in RIPA lysis buffer (iNtRON) with a proteinase inhibitor (Roche), followed by incubation with streptavidin–agarose beads (Pierce) and western blotting. Immunoreactivity was determined by enhanced chemiluminescence (GE Healthcare). The images were captured using a bioimaging analyzer (LAS-3000; Fuji, Tokyo, Japan) and analyzed with the Multi-Gauge program (Fuji, Tokyo, Japan).

### Behavior analysis

Contextual fear conditioning was performed with male mice between 15 and 16 weeks of age, including 8 CBKO mice. We modified the protocol of McHugh et al.^34^ to conduct the contextual fear discrimination task. Mice were trained to discriminate between two similar contexts, A and B, through repeated experience in each context. Context A (the conditioning context) was a chamber (18 cm wide × 18 cm long × 30 cm high; H10-11M-TC; Coulbourn Instruments 5583, PA 18052, USA) consisting of a metal grid floor, aluminum sidewalls, and a clear plexiglass front door and back wall. Context A was indirectly illuminated with a 12 W light bulb. The features of Context B (the safe context) were the same as those of Context A, except for a unique scent (1% acetic acid), a dimmer light (50% of A), and a 15° slope in the floor. Each context was cleaned with 70% ethanol before the animals were placed in. On the first 3 days (contextual fear acquisition), the mice were placed in Context A for 3 min to explore the environment and then received a single footshock (0.75 mA, for 2 s). The mice were returned to their home cage 1 min after the shock. On days 4–5, mice of each genotype were divided into two groups: one group was exposed to Context A on day 4 and Context B on day 5, while the other group was exposed to Context B on day 4 and Context A on day 5. On days 4–5 (generalization), neither group received a shock in Context A or B, and the level of freezing behavior was measured for 5 min only in Context A. We defined freezing behavior as behavioral immobility except for respiratory movement^35^. We observed videos for 2 s bouts every 10 s (18 or 30 observation bouts for 3 min or 5 min of recording time) and counted the number of 2 s bouts during which the mouse displayed freezing behavior (referred to as a freezing score). The percentage of freezing was calculated by dividing the freezing score by the total number of observation bouts (18 or 30). The mice were subsequently trained to discriminate between these two contexts by being placed in the two contexts daily for 8 days (from day 6 to day 13, discrimination task). The mice always received a footshock 3 min after being placed in Context A but not B. Discrimination ratios were calculated as F_A_/(F_A_ + F_B_), where F_A_ and F_B_ are the freezing scores in Contexts A and B, respectively. All experiments and analyses were performed while blinded to the genotypes of the mice.

For one trial of contextual fear conditioning, an experiment was performed with male mice between 14 and 19 weeks of age (eight control mice and eight CBKO mice) in a pair of very distinct contexts (A and C). The aforementioned Context A was used as the conditioning context. The distinct context (Context C) was a white acrylic cylinder with a blind end (15 cm in diameter, 18 cm in height, and 0.5 cm in thickness) standing vertically on the metal grid floor of Context A, and the bottom of the cylinder was covered with cage bedding, on which the mice were placed. The chamber and cylinder were cleaned using 70% ethanol between runs. On day 1 (acclimation), mice were placed in Context A and then placed in Context C an hour later. Mice were allowed to freely explore in both contexts for 5 min. On day 2 (conditioning), all groups of mice were placed in Context A and received a single footshock (0.75 mA, for 2 s) 3 min later. Mice were left in Context A for 1 min after the shock. On day 3 (assessment), mice were separated into two groups; the mice of each group were placed in Context A or C for 3 min without footshock, during which their freezing scores were measured.

An open-field exploration test was used to assess locomotor activity^36^. Open-field exploration was performed with male mice aged 16 weeks (six control mice and six CBKO mice). Mice were handled before the open-field exploration behavior test (OFT). Mice were picked up by the tail and supported without restraint in the palm of an experimenter’s hand for 15 min/day for 4 days. Following handling, each mouse was placed in a holding cage until all mice had been handled. They were then returned to their home cage. During mouse handling, handlers wore latex gloves, which they changed between mice. After the handling day, we executed the OFT using a modified version of the protocol of Kim et al.^36^. The open-field box was made of white plastic (40 × 40 × 40 cm) and divided into a center zone (20 × 20 cm) and an outer field. The entire field was illuminated with diffuse light, and white noise was played in the background. Individual mice were placed in the center zone, and the path of each animal was recorded with a video camera. In a 20 min of observation period, the total distance traveled during the total period and the time spent in the center zone in the initial 5 min were analyzed using the program EthoVision XT (Noldus, Virginia, USA).

### Statistical analysis

Data were analyzed with IgorPro (Version 6; Wave-Metrics, Lake Oswego, USA) and are presented as the mean ± SEM with the number of cells or mice (*n*) used in each experiment. The statistical significance was evaluated using Student’s *t* test, and the level of significance is indicated by the number of asterisks (*P < 0.05; **P < 0.01; ***P < 0.001). *P* > 0.05 was regarded as not significantly different (N.S.). Data from behavioral studies were analyzed using IgorPro and Origin (Version 8; Microcal, Northampton, USA). Comparisons between multifactorial statistical data were made using a two-way analysis of variance (ANOVA). The differences in time-dependent changes in behavioral parameters between the two genotypes were evaluated using two-way repeated-measures ANOVA.

## Results

### Increased excitability in the CBKO GCs

**Fig. 1:**
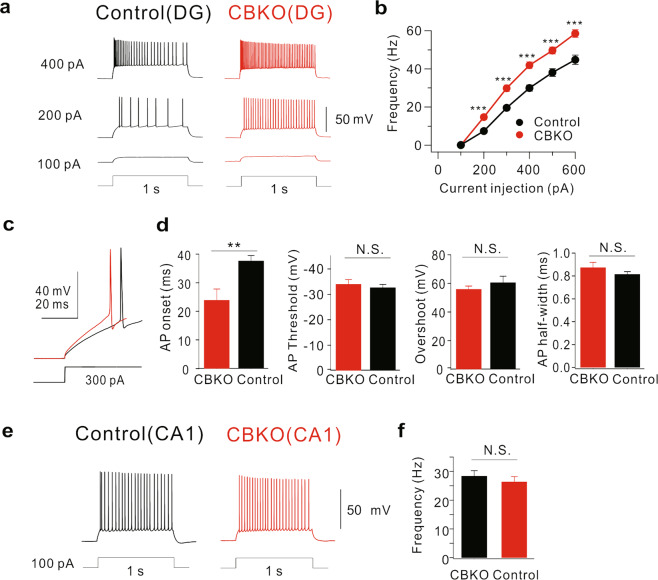
Increased firing frequency in dentate GCs of CBKO mice. **a** Trains of APs induced in dentate GCs of control (black) and CBKO mice (red) by 1 s depolarizing current injections as indicated by numbers left to the traces. **b** The F–I curves in the control (black, *n* = 9) and CBKO (red, *n* = 8) mice. **c** Example traces of the first AP elicited by 300 pA current injection from control (black) and CBKO (red) GCs. **d** Bar graphs showing the onset time, threshold, overshoot amplitude, and half-width of the first AP elicited by 300 pA current injection measured from CBKO (red) and control (black). **e** Trains of APs induced in CA1 of control (black) and CBKO (red) by 100 pA, 1 s depolarizing current injection. **f** Bar graph for AP frequency at 100 pA current injection in CA1 cells of control and CBKO mice. Data are represented as the mean ± SEM. **P < 0.01, ***P < 0.001, N.S. (not significant) *P* > 0.05 by Student’s *t* test.

Reduced Ca^2+^ buffering may affect the Ca^2+^ homeostasis of cells. However, the relationship between the extent of Ca^2+^ buffering and the specific changes in neuronal function is not well-characterized. Using CBKO mice, we tested the possibility that Ca^2+^ dysregulation caused by reduced Ca^2+^ buffering could alter neuronal excitability in mature GCs of the DG, where CB is a major Ca^2+^ buffer^3^. To avoid the effect of other Ca^2+^ buffers in neural progenitor cells and immature newborn neurons, we chose mature GCs located in the outer granular cell layer that had an input resistance (R_in_) lower than 200 MΩ. We confirmed that the average resting membrane potential (RMP; −81.6 ± 1.8 mV, *n* = 9, for control GCs; −84.4 ± 1.0 mV, *n* = 8 for CBKO GCs, *P* = 0.198) and R_in_ values (145.1 ± 11.4 MΩ for control GCs; 159.1 ± 13.4 MΩ for CBKO GCs, *P* = 0.439) were not significantly different between control and CBKO GCs. Interestingly, we found that the firing frequency in response to long depolarizing pulses was significantly higher in the CBKO GCs (red, Fig. 1a) than in the control GCs (black, Fig. 1a). As a measure of neuronal excitability, we plotted the firing frequencies (F) against the amplitude of the injected currents (I). The F–I relationship was steeper in the CBKO genotype (Fig. 1b), suggesting that abnormal Ca^2+^ homeostasis caused by reduced Ca^2+^ buffering induces ion channel remodeling in the GCs, which, in turn induces hyperexcitability. The increased firing frequency was accompanied by a shortening of the onset time of action potentials (APs; 37.7 ± 1.8 ms, *n* = 8 for control; 23.9 ± 3.9 ms, *n* = 6 for CBKO GCs, *P* = 0.01388, Fig. 1c, d). The AP threshold and AP shapes, measured by overshoot and half-width, were not affected in the CBKO GCs (Fig. 1d). In contrast, no significant difference was found in CA1 pyramidal neurons between control and CBKO mice (Fig. 1e, f), suggesting that the changes in ion channels that cause hyperexcitability in the CBKO genotype are specific to GCs.

### Impaired pattern separation in CBKO mice

**Fig. 2:**
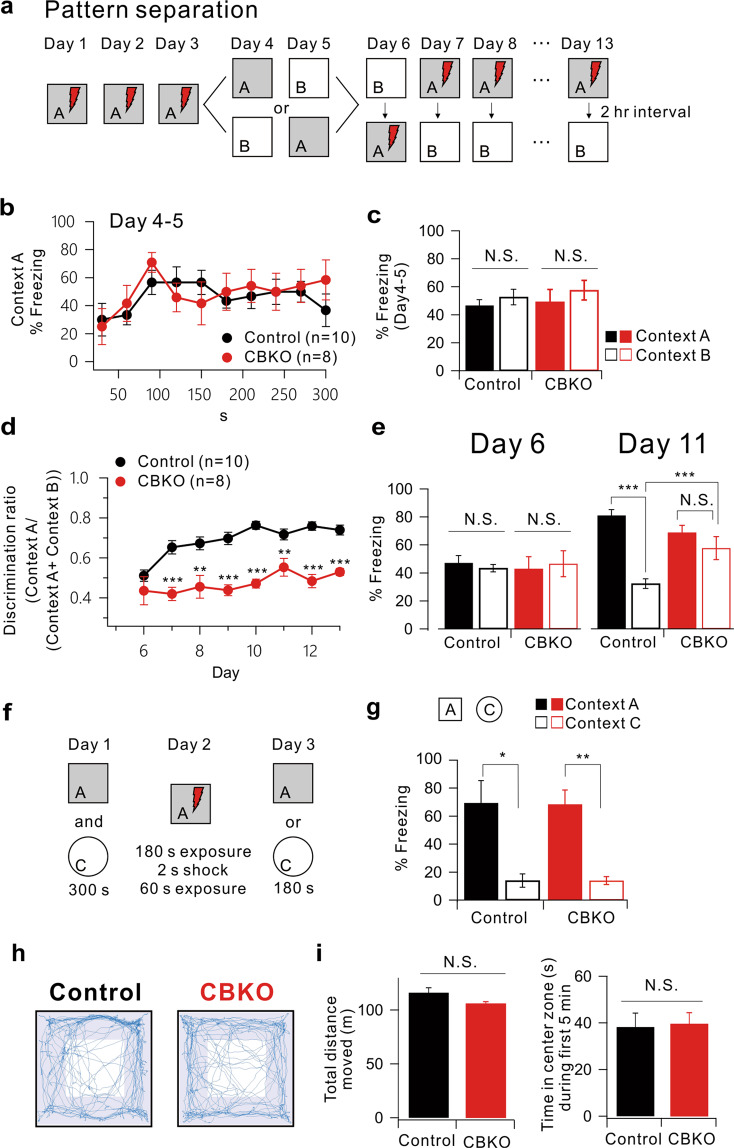
Impaired pattern separation in CBKO mice. **a** Experimental procedure for pattern separation in 15- to 19-week-old control (*n* = 10) and CBKO (*n* = 8) mice. **b** On days 4 and 5, the kinetics of freezing across the 5 min test in Context A. **c** The percentage of freezing in Context A (filled bar) and Context B (open bar) on days 4 to 5 in both contexts (A and B). Control (black, *n* = 10) and CBKO (red, *n* = 8) mice displayed equal amounts of freezing in both contexts (A and B). **d** On days 6–13, the time course of the discrimination ratio in control (black, *n* = 10) and CBKO (red, *n* = 8) mice. **e** The percentage of freezing in Context A (filled bar) and Context B (open bar) for the control (black, *n* = 10) and CBKO (red, *n* = 8) mice on day 6 (left) and day 11 (right). **f** Experimental procedure for one-trial contextual fear conditioning between control (*n* = 8) and CBKO (*n* = 8) mice. **g** The percentage of freezing in Context A (filled bar) and context C (open bar, distinct object) for the control (*n* = 8) and CBKO (*n* = 8) mice. **h** The trajectories traveled by the control (left) and CBKO (right) mice in the open-field test showed how the mice traveled. **i** Summary bar graph of the total traveled distance (left) and time in the center zone during the first 5 min (right). Total distance moved (m), Control, *n* = 6, 116.20 ± 4.45, CBKO, *n* = 6, 106.11 ± 1.79, *P* = 0.06175; Time in the center zone (sec), Control, *n* = 6, 11.81 ± 1.22, CBKO, *n* = 6, 13.91 ± 1.55, *P* = 0.31208. Data are represented as the mean ± SEM.

Low excitability of the DG is considered important for information processing in the hippocampus, particularly for pattern separation^37^. We addressed whether the increased firing of GCs in CBKO mice leads to an impairment in pattern separation during the contextual fear discrimination test. We first subjected CBKO mice to contextual fear conditioning using a pair of similar contexts (A and B). Both had the same metal grid floor, but B had a unique odor (1% acetic acid), dimmer lighting (50% of A), and a 15° slope on the floor. As shown in Fig. 2a, the mice learned to discern a similar context (Context B) over several days with a single footshock in Context A. On the first 3 days, mice were placed only into A, receiving a single footshock after 180 s. On days 4 and 5, the mice of each genotype were divided into two groups. One group of each genotype was exposed to Context A on day 4 and to Context B on day 5, while the other group was exposed to Context B on day 4 and to Context A on day 5. No group received a footshock in either context, and freezing was evaluated for 5 min. Both genotypes showed identical freezing kinetics across the 5 min test in Context A (Fig. 2b) and equivalent generalization between contexts (Fig. 2c, two-way ANOVA, genotype: *F*_(1,32)_ = 0.30, *P* = 0.58, context: *F*_(1,32)_ = 1.17, *P* = 0.29, genotype × context: *F*_(1,32)_ = 0.03, *P* = 0.86). The mice were subsequently trained to discriminate these contexts by visiting the two contexts daily for 8 days in 2-h intervals (from day 6 to 13), always receiving a footshock 180 s after being placed in Context A but not in Context B. The daily discrimination ratio was calculated as the ratio of freezing during the 180 s in Context A to the total freezing during both visits (A and B). On day 6, neither genotype could distinguish between contexts (Fig. 2d, e, genotype: *F*_(1,32)_ = 0.005, *P* = 0.94, context: *F*_(1,32)_ = 0.001, *P* = 0.97, genotype × context: *F*_(1,32)_ = 0.32, *P* = 0.57); thus, the discrimination ratio was ~0.5. As the experiment progressed, the control mice gained the ability to discriminate Context B from Context A effectively, and the discrimination ratio increased. However, CBKO mice exhibited significant deficits in the acquisition of discrimination ability (Fig. 2d) and showed elevated freezing in the shock-free Context B (Fig. 2e, *t* test, Context B, *P* < 0.0001, two-way ANOVA, genotype: *F*_(1,32)_ = 6.71, *P* = 0.01, context: *F*_(1,32)_ = 52.99, *P* < 0.0001, genotype × context: *F*_(1,32)_ = 25.60, *P* < 0.0001). To examine whether the impaired discrimination between similar contexts in CBKO mice was due to a problem with memory acquisition, we examined the context specificity of the conditioning by assessing freezing behavior using a distinct pair of contexts (A and C). This distinct context (context C) evoked significantly lower levels of freezing (similar in the two genotypes) than Context A (Fig. 2f, g, genotype: *F*_(1,11)_ = 0.002, *P* = 0.96, context: *F*_(1,11)_ = 30.34, *P* < 0.0001, genotype × context: *F*_(1,11)_ = 0.002, *P* = 0.96). These data imply that CBKO mice have no deficit in learning or discriminating between very distinct contexts but have difficulty discriminating between similar contexts (pattern separation). We also tested the locomotor activity and anxiety levels of CBKO mice using the open-field test. Control and CBKO mice showed similar exploratory patterns, as they moved the same distance in the open-field box and spent a similar amount of time in the center zone (Fig. 2h, i).

### A reduced Kv4.1 current underlies increased excitability in CBKO GCs

**Fig. 3:**
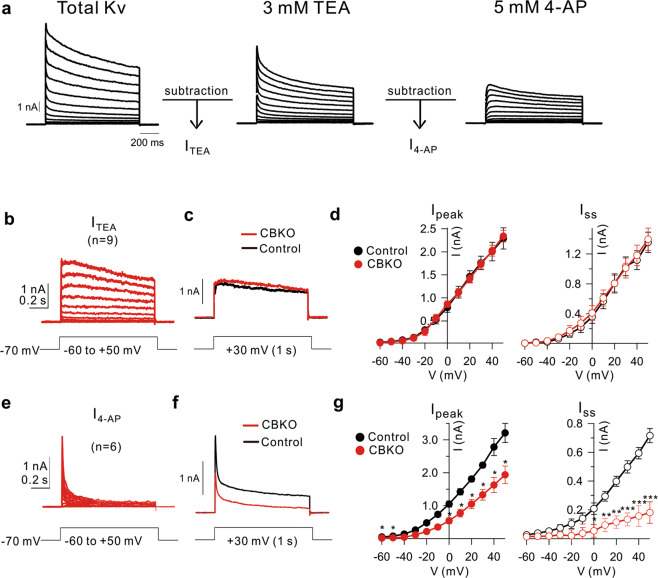
Decrease 4-AP-sensitive current in CBKO GCs. **a** Representative whole-cell voltage-gated K^+^ currents from control dentate GCs. Currents were evoked in response to 1 s, 10 mV voltage steps to potentials between −60 and +50 mV from a holding potential of −70 mV. After recording the total outward currents, we changed the bath solution, which contained 3 mM TEA, and the difference currents before and after TEA were measured to obtain TEA-sensitive currents (I_TEA_). Subsequently, 5 mM 4-AP was added to obtain 4-AP-sensitive currents (I_4-AP_). **b** I_TEA_ obtained from CBKO GCs (*n* = 9) were averaged. **c** Superimposed I_TEA_ traces at +30 mV measured CBKO (red) and control (black) at +30 mV. **d** Amplitudes of the peak currents (I_peak_) and steady-state currents (I_ss_) for I_TEA_ are plotted as a function of the given potential (V). Control (black closed circle, *n* = 4) and CBKO (red closed square, *n* = 9). **e** The difference currents before and after applying 5 mM 4-AP. **f** Superimposed I_4-AP_ traces measured from CBKO (red) and the control (black) at +30 mV. **g** I_peak_ and I_ss_ for 5 mM 4-AP-sensitive I_A_ currents, as shown in Fig. 3d. Control (black closed circle*, n* = 9) and CBKO (red closed circle, *n* = 6). Data are represented as the mean ± SEM. *P < 0.05, **P < 0.01, ***P < 0.001, N.S. not significant (*P* > 0.05) by Student’s *t* test.

We then investigated the ion channel mechanisms responsible for the hyperexcitability of CBKO GCs. Since the increased firing was not associated with a change in the AP threshold, the involvement of Na^+^ channels was unlikely. Therefore, we analyzed the difference in K^+^ currents between CBKO and control CGs. Whole-cell K^+^ currents were recorded in voltage-clamp mode by applying depolarizing voltage steps from −60 to +50 mV (in 10 mV increments, 1 s duration) from a holding potential of −70 mV. TTX, Cd^2+^/Ni^2+^, bicuculline, and CNQX were added to the external solution to block Na^+^ channels, Ca^2+^ channels, GABA_A_ receptors, and AMPA/kainate receptors, respectively. Total K^+^ currents were dissected into TEA-sensitive (I_TEA_) and 4-AP-sensitive K^+^ currents (I_4-AP_) (Fig. 3a). The I_TEA_ obtained from CBKO GCs (*n* = 9) showed no significant difference from that of control GCs, as reported previously^25^; both the peak (I_peak_ at +30 mV; 1.807 ± 0.152 nA, *n* = 9, for control; 1.727 ± 0.096 nA, *n* = 9, for CBKO, *P* = 0.66) and steady-state currents (I_ss_ at +30 mV; 1.107 ± 0.126 nA, *n* = 9, for control: 1.012 ± 0.133 nA, *n* = 9, for CBKO, *P* = 0.609) were comparable between genotypes (Fig. 3b–d). In contrast, the characteristics of I_4-AP_ in CBKO GCs were different from those in control GCs. The inactivation phase of I_4-AP_ in CBKO GCs showed a fast time constant with little I_ss_ (Fig. 3e), which are typical characteristics of A-type K^+^ currents, while that in the control GCs had a significant proportion of I_ss_, which is attributable to Kv4.1 currents (Fig. 3f)^25^. The amplitude of I_4-AP_ in CBKO GCs was significantly smaller than that in the control GCs (Fig. 3g). The reduction in I_ss_ (63%) was more pronounced than the reduction in I_peak_ (36%). I_4-AP_ in CBKO GCs was similar to I_4-AP_ in the control GCs in the presence of anti-Kv4.1 antibodies, as reported previously^25^. This suggests that the outward K^+^ current component reduced in the CBKO GCs is likely to be the Kv4.1 current.

### Calbindin regulates the cell-surface localization of Kv4.1 via CaMKII-mediated phosphorylation

**Fig. 4:**
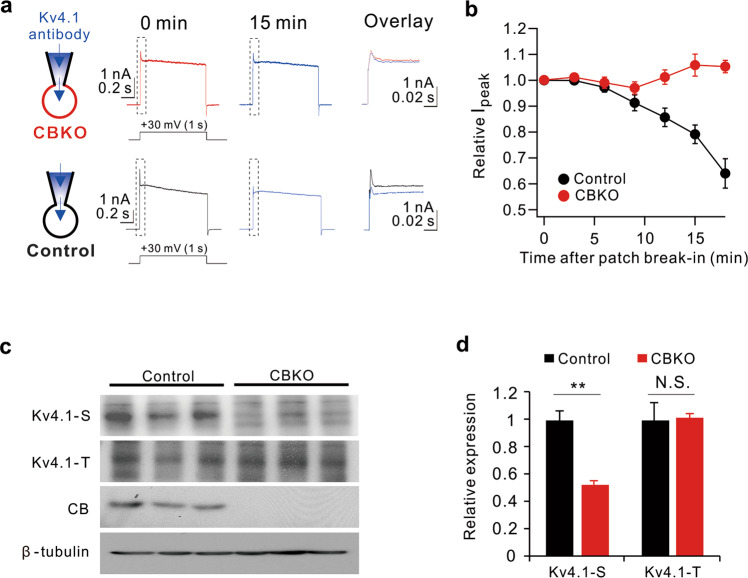
Evidence for the reduced Kv4.1 currents in CBKO GCs. **a** Top: whole-cell, voltage-gated K currents activated by +30 mV depolarizing pulses in CBKO GCs with intracellular solution containing Kv4.1 antibodies, recorded shortly after rupture (0 min) and after full diffusion (15 min). Each trace is expanded and superimposed for comparison (dashed box). Bottom: same as described at (top) but in control GCs. **b** Time course of normalized I_peak_ at each time point after break-in. **c** Western blot analysis of total Kv4.1 (Kv4.1-T) and surface Kv4.1 (Kv4.1-S) in the DG of 8-week-old control and CBKO mice. The absence of calbindin (CB) expression was confirmed in the DG of CBKO mice. β-Tubulin was used as a loading control. **d** Relative protein levels of Kv4.1 in surface or total lysates in CBKO cells compared to control cells. Values are means of three determinants ± SD. (*n* = 3). **P < 0.01, N.S. not significant by Student’s *t* test.

To further confirm that the K^+^ current component reduced in CBKO GCs is the Kv4.1 current, we monitored changes in the outward current amplitude during perfusion of anti-Kv4.1 antibodies through a patch pipette after patch break-in. Anti-Kv4.1 antibodies significantly reduced the outward K^+^ currents in the GCs of control mice but did not induce changes in CBKO GCs (Fig. 4a, b), suggesting that Kv4.1 is not functional in CBKO GCs.

To investigate the mechanism of reduced Kv4.1 current in CBKO GCs, we first tested alterations in Kv4.1 protein expression and surface localization. To monitor membrane localization, isolated DG was labeled with biotin, lysed, and subjected to pulldown with streptavidin beads. Immunoblot analysis revealed that the level of surface-resident Kv4.1 (Kv4.1-S) was significantly reduced in the CBKO DG, while the level of total Kv4.1 protein (Kv4.1-T) was not significantly altered relative to the control DG (Fig. 4c, d). These results suggest that the reduced Kv4.1 current in CBKO GCs is attributable to the impairment of Kv4.1 trafficking to the membrane surface.

**Fig. 5:**
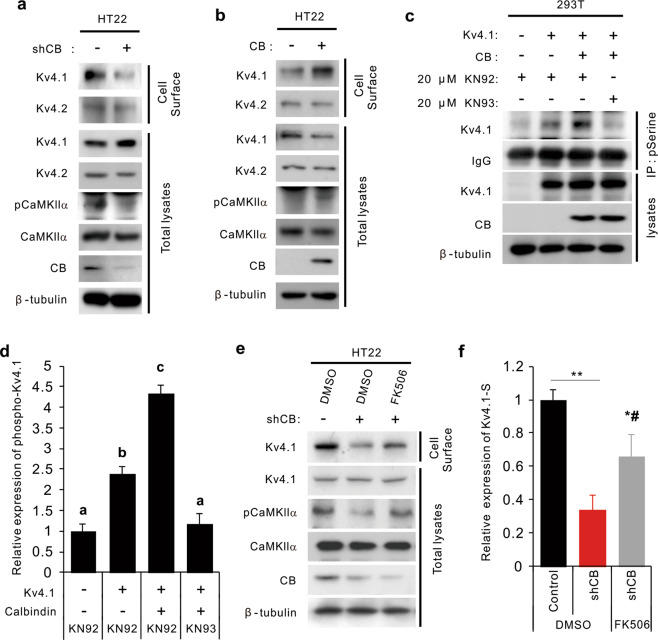
Calbindin regulates the cell-surface localization of Kv4.1 via CaMKII-mediated phosphorylation. **a**, **b** Cell-surface biotinylation assay of control and shRNA calbindin (**a**) or calbindin (**b**)-transfected HT22 cells followed by immunoblot analysis with the indicated antibodies. **c** Analysis of serine phosphorylation of Kv4.1 in 293T cells expressing control or Kv4.1 and calbindin treated with 20 µM KN-92 or KN-93 for 24 h. **d** Quantification of the relative phosphorylation levels of Kv4.1 at serine residues shown in (**c**). The intensities were normalized to the level of β-tubulin. Values are means of triplicate determinants (ANOVA Tukey). Letters indicate statistically distinct groups (*P* < 0.05). **e** Surface biotinylation assay of control and calbindin-depleted HT22 cells treated with vehicle or 10 µM FK506 for 1 h followed by immunoblot analysis with the indicated antibodies. **f** Quantification of the relative cell-surface protein levels of Kv4.1 shown in (**e**). The intensities were normalized to the level of β-tubulin. *P < 0.05, **P < 0.01 versus the control group; ^#^*P* < 0.05 versus the shCB group.

To further understand the mechanism underlying changes in Kv4.1 trafficking by reduced CB, we infected the mouse hippocampal neuronal cell line HT22 with lentiviruses expressing either a control scrambled RNA or CB-shRNA and examined the surface expression of Kv4.1 in differentiated cells. Similar to the results obtained with the CBKO DG, the level of membrane-resident Kv4.1 protein was specifically reduced in CB-depleted HT22 cells without any reduction in the total Kv4.1 level (Fig. 5a). We then examined the level of active phosphorylated CaMKIIα (p-CaMKIIα), which acts as the main player in the Ca^2+^ signaling pathway implicated in diverse cellular processes, including the control of neuronal excitability^38^. The level of p-CaMKIIα was substantially reduced in CB-depleted HT22 cells (Fig. 5a). Conversely, CB overexpression enhanced the levels of surface Kv4.1 and p-CaMKIIα relative to those of the control group (Fig. 5b). These data suggest that CaMKIIα might be a downstream effector of CB to control Kv4.1 surface localization, likely through phosphorylation. Thus, we investigated the potential phosphorylation of Kv4.1 in relation to CB levels and CaMKII activities. HEK293T cells were transfected with the expression vectors for Kv4.1-GFP with or without CB, and 2 days later, the cells were treated with the CaMKII inhibitor KN-93 or the relevant control compound KN-92 for 24 h. To monitor the level of phosphorylated Kv4.1, cell lysates were subjected to immunoprecipitation with anti-phosphoserine antibodies and immunoblotting analysis. Coexpression of CB and Kv4.1 significantly elevated the phosphorylated Kv4.1 protein level in cells treated with the control compound KN-92, and this effect was abrogated by KN-93 treatment (Fig. 5c, d). These data suggest that CB facilitates the CaMKII-mediated phosphorylation of Kv4.1, thereby increasing its cell-surface expression in DG and hippocampal cells.

Since the Ca^2+^ buffering capacity of CBKO GCs is only half that of control GCs^3^, the intracellular Ca^2+^ concentration is expected to be higher in CBKO GCs (CBKO GCs are subject to Ca^2+^ overload). Thus, it appears to be paradoxical that CaMKII inhibition shows a phenotype similar to that of CBKO. To understand how p-CaMKII is downregulated by CB knockdown, we analyzed the effect of a Ca^2+^-dependent phosphatase inhibitor, FK506, on p-CaMKII and the surface localization of Kv4.1 in CB-depleted HT22 cells (Fig. 5e, f). CB depletion reduced the level of surface Kv4.1 protein after treatment with the vehicle dimethyl sulfoxide (DMSO), while FK506 treatment significantly restored the surface Kv4.1 levels (Fig. 5e, f). These results suggest that CB knockdown may activate Ca^2+^-dependent phosphatase, which, in turn, decreases p-CaMKII levels and Kv4.1 surface localization.

### Blockade of CaMKII induces hyperexcitability in GCs

**Fig. 6:**
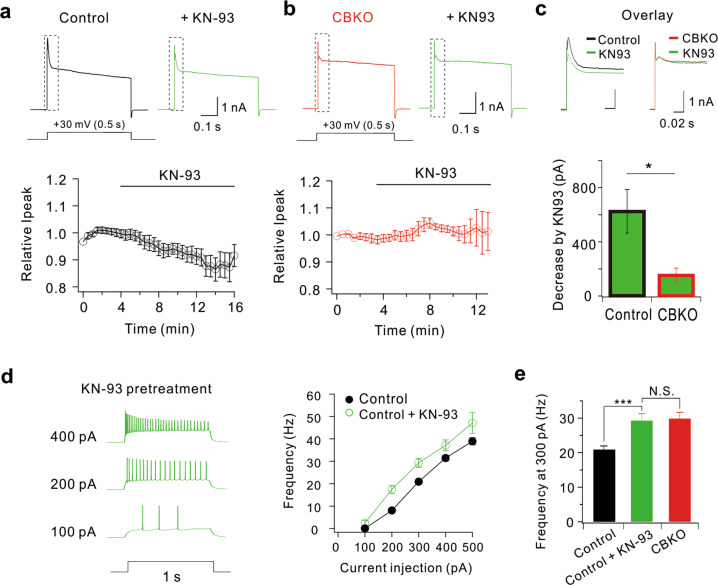
KN-93 decreases K^+^ currents in control GCs but not in CBKO GCs. **a** Representative whole-cell K^+^ currents at + 30 mV in control GCs before (black) and after KN-93 application (green). Changes in K^+^ current amplitude after KN-93 application were averaged from seven cells, and the mean ± SEM was plotted (lower panel). **b** Representative whole-cell K^+^ currents at +30 mV in CBKO GCs before (red) and after KN-93 application (green). Changes in K^+^ current amplitude after KN-93 application were averaged from seven cells, and the mean ± SEM was plotted (lower panel). **c** Representative whole-cell K^+^ currents at +30 mV shown in (**a**, **b**) with dotted boxes are overlaid on an expanded time scale. Bar graphs indicate decreases in I_peak_ by KN-93 (lower panel). Mean ± SEM. **d** Representative AP traces induced by injecting depolarizing currents as indicated by the number left to the traces in control GCs pretreated with KN-93 (left) and the corresponding F–I curve (green circles, right). The F–I curve from control GCs shown in Fig. 1b was superimposed for comparison (black line). **e** The firing frequency obtained by 200 pA current injection obtained in the presence of KN-93 in control GCs (green, *n* = 7) was compared with that in the control (black), as shown in Fig. 1b. Data are represented as the mean ± SEM. ***P < 0.001. N.S. not significant by Student’s *t* test.

To confirm whether CaMKII-dependent phosphorylation is a key mechanism for Kv4.1 activity in GCs, we examined the effect of a CaMKII inhibitor (KN-93) on outward K^+^ currents (Fig. 6a). In control GCs, outward K^+^ currents were reduced significantly by KN-93 (625.6 ± 160.9 pA at +30 mV, *n* = 7), which was comparable to the previously reported Kv4.1 current amplitude in control GCs (619.6 ± 66.4 pA)^25^. In contrast, KN-93 did not show a significant effect in CBKO GCs (Fig. 6b, c), which was consistent with the idea that the CaMKII-dependent Kv4.1 current is absent in CBKO GCs. Accordingly, GCs pretreated with CaMKII inhibitors displayed hyperexcitability, which was comparable to that of CBKO GCs (Fig. 6d, e). These results support the idea that the same outward K^+^ current component decreased by CaMKII inhibitors is also reduced in CBKO GCs.

### CaMKII regulates Kv4.1 activity by phosphorylating S555 of Kv4.1

**Fig. 7:**
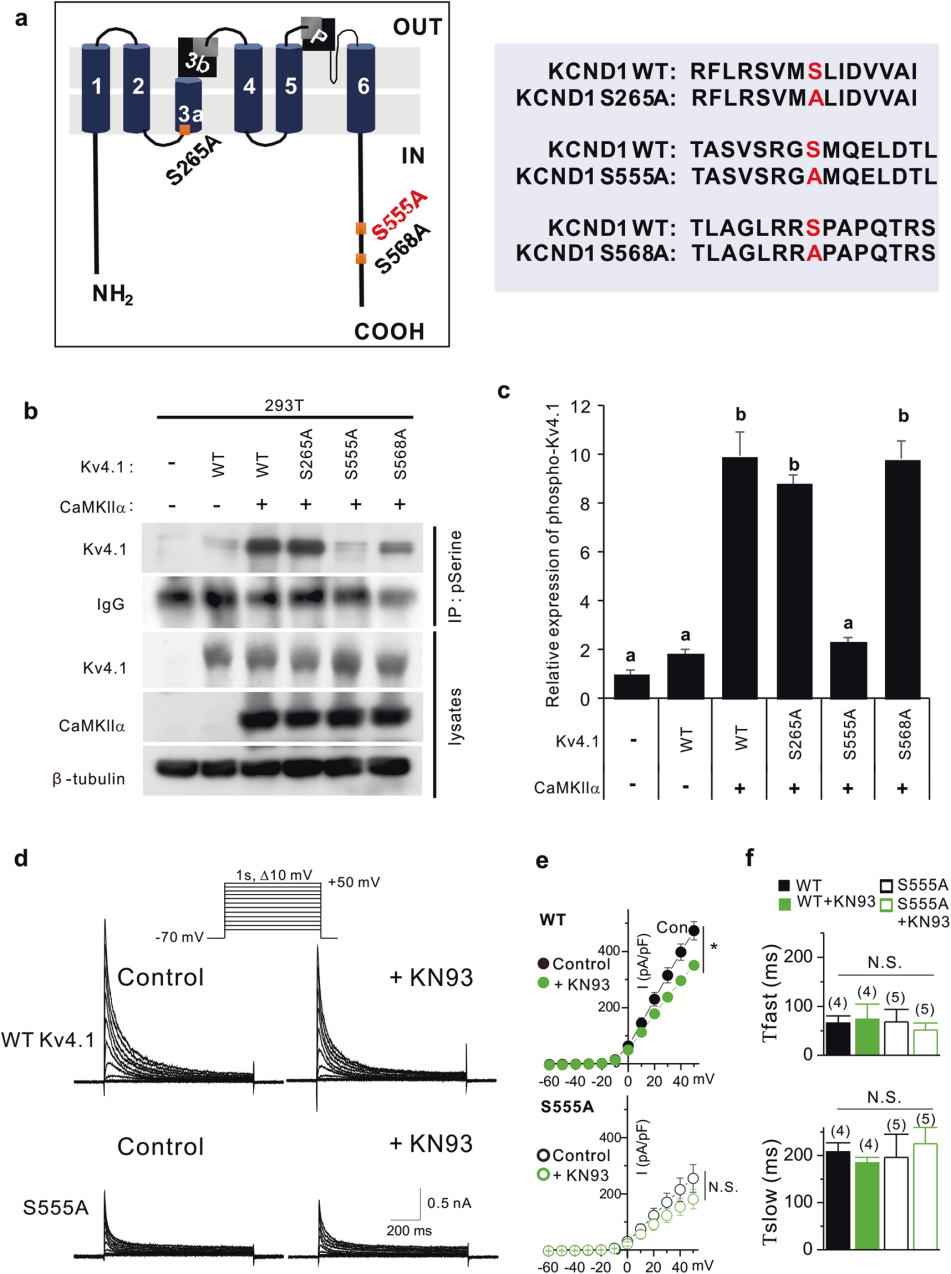
CaMKII regulates Kv4.1 activities by phosphorylating S555 of Kv4.1. **a** Schematic representation of the domain structure of Kv4.1. Potential serine phosphorylation sites are labeled red. (left) Alignments of the potential serine phosphorylation site are shown in red, and serine residues (S) are switched to alanine (A). **b** Immunoprecipitation with anti-phospho-serine antibody and immunoblotting with the indicated antibodies in 293T cells expressing control, WT Kv4.1, or mutant Kv4.1 (S265A, S555A, and S568A) with CaMKII. **c** Quantification of serine phosphorylation levels of Kv4.1. Values are the means of triplicate determinants (ANOVA Tukey, *P* < 0.05). Letters indicate statistically distinct groups. **d** Representative current recordings from HEK293T cells expressing WT Kv4.1 (upper) or S555A Kv4.1 (lower) before and after KN-93 (1 μM) treatment for 5 min. Currents were elicited by voltage steps from −60 to +50 mV at a holding potential of −70 mV. **e** Current–voltage curves for peak current amplitude of WT and S555A Kv4.1 currents in the absence and presence of KN-93 (1 μM). Data represent mean ± SEM. *P < 0.05, Paired Student’s *t* test. **f** The effect of S555A mutation and KN-93 on the fast and slow inactivation time course. The inactivation time course of Kv4.1 currents was well fitted with a double exponential function. Data are represented as the mean ± SEM. N.S. not significant by Student’s *t* test.

A sequence analysis to predict the phosphorylation sites in Kv4.1 by CaMKII using the PhosphoSite database revealed three potentially phosphorylated serine residues at positions 265, 555, and 568 of Kv4.1 (Fig. 7a). Thus, we generated serine-to-alanine mutants for these sites in Kv4.1 and analyzed the phosphorylation status by CaMKII activity. Co-transfection with Kv4.1 and CaMKIIα elevated Kv4.1 levels immunoprecipitated with anti-phospho-serine antibody compared to the Kv4.1 single-expression control (Fig. 7b, c). The phosphorylation levels of R265A and S568A mutants were equivalent to wild-type Kv4.1 in response to CaMKIIα. In contrast, the S555A mutant failed to show any increase in response to CaMKIIα, suggesting that S555 is a target phosphorylation site for CaMKIIα.

We further examined the correlation between CaMKII-induced phosphorylation and Kv4.1 channel function by recording whole-cell currents in HEK293T cells expressing WT or S555A Kv4.1 (Fig. 7d, e). Compared to WT, S555A Kv4.1 exhibited remarkably decreased currents. The current density at +50 mV was 473.58 ± 32.3 pA/pF (*n* = 4) for WT Kv4.1, and it decreased to 254.86 ± 49.34 pA/pF (*n* = 5; *P* < 0.05) for S555A Kv4.1. Furthermore, CaMKIIa inhibition with KN-93 decreased WT Kv4.1 activity to 350.36 ± 10.57 pA/pF (*n* = 4, *P* < 0.05 vs. WT Kv4.1 under control conditions), while it did not further reduce S555A Kv4.1 activity (180.92 ± 34.71 pA/pF, *n* = 5; *P* > 0.05 vs. S555A Kv4.1 under control conditions). The S555A mutation and KN-93 treatment did not change the inactivation kinetics (Fig. 7f). These data suggest that the phosphorylation of the S555 residue in Kv4.1 by CaMKII is a key regulatory step in Kv4.1 activity.

### The functional deficit in calcium buffering in Tg2576 GCs is restored by antioxidant treatment

**Fig. 8:**
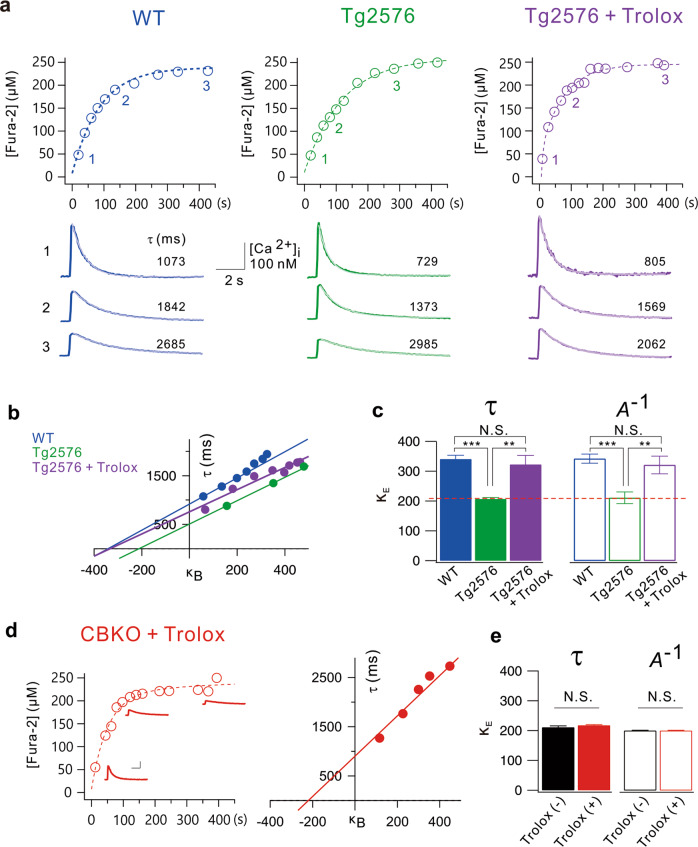
Endogenous Ca^2+^ binding ratios (κ_E_) are reduced in dentate GCs of Tg2576 GCs. **a** Time course of fura-2 loading (250 µM) during whole-cell patch recording in wild-type (WT, blue), Tg2576 (green), and Tg2576 slices pretreated with Trolox (500 μM, violet) for 1 hr. Ca^2+^ transients (CaTs) were evoked by a depolarizing pulse (from −70 to 0 mV, 40 ms in duration). Fura-2 concentrations were calculated from isosbestic fluorescence and plotted as a function of whole-cell recording time. Exemplary CaTs shown underneath the loading curve were obtained at 20, 135, and 427 s in WT, 40, 124, and 282 s in Tg2576, and 9, 103, and 392 s in Tg2576 + Trolox. Inset scale bars indicate 100 nM [Ca^2+^]_i_ and 2 s. **b** Plots of time constants (τ) for the decay phases of CaTs as a function of incremental Ca^2+^ binding ratios of fura-2 (κ_B_). An *x*-intercept of the linear fit to this plot was considered the endogenous Ca^2+^ binding ratio (κ_E_). **c** Mean values for κ_E_ from WT (blue*, n* = 10), Tg2576 (green*, n* = 7) and Tg2576 + Trolox (violet, *n* = 6) dentate GCs estimated from Fig. 7b (solid bars). The κ_E_ values were also estimated from the plot of 1/amplitude versus κ_B_ (open bars). The dashed line (red) indicates the κ_E_ value of CBKO GCs reported previously^3^. **d** (left) Time course of fura-2 in CBKO slices pretreated with Trolox for 1 hr. Scale bar: 100 nM [Ca^2+^], 2 s. (right) Plots of time constants for the decay phases of CaTs as a function of incremental Ca^2+^ binding ratios of fura-2 (κ_B_). **e** Mean values for κ_E_ from CBKO with or without Trolox dentate GCs. The κ_E_ values of Trolox(−) were adapted from a previous study^3^, and those of Trolox(+) were estimated from Fig. 7d. Data are represented as the mean ± SEM. **P < 0.01, ***P < 0.001, N.S. not significant by Student’s *t* test.

Neuronal depletion of CB was reported to be tightly linked to AD-related cognitive deficits. However, this depletion was observed in AD model mice aged at least 6 months^8^. We recently reported that Kv4.1 is downregulated, leading to hyperexcitability in dentate GCs in 1–2-month-old Tg2576 mice^39^. Therefore, we asked whether dysfunctional Ca^2+^ buffering has pathophysiological significance in early preclinical stages. To this end, we measured the cellular Ca^2+^ buffering capacity in mature GCs from 1- to 2-month-old WT and Tg2576 mice using a method described in our previous paper^3^. To estimate κ_E_ (the sum of the Ca^2+^ binding ratio of mobile and static endogenous Ca^2+^ buffers), we loaded GCs with fura-2 (250 µM) using whole-cell patch pipettes (R_S_ = 20.5 ± 1.0 MΩ), and evoked Ca^2+^ transients (CaTs) by applying voltage pulses (from −70 to 0 mV, 40 ms duration) at different time points during fura-2 loading (Fig. 8a). As the cytosolic fura-2 concentration increased gradually after patch break-in, CaTs exhibited decreased amplitudes (*A*) and increased decay time constants (τ). To estimate the κ_E_ value, we plotted τ against κ_B_ and obtained the *x*-intercept from the extrapolation of the linear fit (Fig. 8b). The mean κ_E_ value in WT GCs estimated from the τ vs κ_B_ plots was 340.7 ± 12.5 (*n* = 10), while that in Tg2576 GCs was 206.8 ± 4.7 (*n* = 7), which was significantly lower than that in WT GCs (*P* = 0.00000034, Fig. 8c). The mean κ_E_ values estimated from the *A*^−1^ vs κ_B_ plots showed similar results (WT GCs: 342.4 ± 15.4, *n* = 10, Tg2576 GCs: 211.4 ± 19.4, *n* = 7, *P* = 0.000081, Fig. 8c). Notably, the κ_E_ value estimated in Tg2576 GCs is comparable to that in CBKO GCs reported previously (210.9 ± 4.8, red dashed line, Fig. 8c)^3^. These results suggest that reduced Ca^2+^ buffering in Tg2576 GCs may be attributable to impaired CB function.

CB has two N-terminal cysteine residues that undergo redox-driven structural changes, and oxidized CB has a lower Ca^2+^ binding affinity than reduced CB^40^. We previously showed that mitochondrial reactive oxygen species (ROS) production is increased in the dentate GCs of 1- to 2-month-old Tg2576 mice, leading to depolarization of the mitochondrial potential, which was reversed by the antioxidant Trolox^26^. To test the possibility that oxidative stress induces the oxidation of CB to compromise the Ca^2+^ buffering capacity, we examined whether the decreased κ_E_ in Tg2576 GCs was restored by antioxidant treatment. When hippocampal slices obtained from Tg2576 mice were incubated in Trolox (500 µM) for 1 h, the κ_E_ value of Tg2576 GCs was 322.98 ± 30.2 (violet, estimated from the τ vs κ_B_ plots, Fig. 8a), which was not significantly different from the κ_E_ value of WT GCs (blue, Fig. 8a, *P* = 0.53941). To confirm whether the effect of Trolox on κ_E_ in the Tg2576 GCs was indeed attributable to the protection of calbindin from oxidation, we tested the effect of Trolox on κ_E_ in the CBKO GCs. Incubation of hippocampal slices obtained from CBKO mice with Trolox treatment for 1 h did not affect κ_E_ in CBKO GCs (Fig. 8d, e), suggesting that Trolox did not have an impact on Ca^2+^ buffer capacity originating from Ca^2+^ buffers other than calbindin. Reduced Ca^2+^ buffering and its restoration by Trolox in Tg2576 GCs suggest that CB function is impaired by oxidative stress.

## Discussion

The roles of endogenous Ca^2+^ buffers in the brain have been investigated mostly by analyzing the phenotypes of mice either lacking or overexpressing one of the Ca^2+^ buffers^1^. Mice with reduced CB expression show deficits in memory and hippocampal long-term potentiation^41^, reduced short-term facilitation^42^, and impaired motor coordination^1,43^. In contrast, the overexpression of CB in DG GCs was shown to alter mossy fiber presynaptic function and impair hippocampal-dependent memory^44^. These data suggest that proper levels of CB are critical for neuronal activity control. However, the mechanisms underlying the involvement of CB in these phenotypes are largely unknown. In this study, we investigated the cellular and molecular mechanisms underlying the excitability changes in dentate GCs in CBKO mice and found that Ca^2+^ dysregulation caused by the loss of CB induces Kv4.1 downregulation, leading to hyperexcitability in dentate GCs. In addition, we observed impaired pattern separation in CBKO mice. These results, together with previous results showing that mice with selective depletion of Kv4.1 in the DG exhibit impaired pattern separation^25^, highlight the importance of the low excitability of dentate GCs in pattern separation.

CB loss in dentate GCs has been shown to be tightly linked to AD-related cognitive deficits^8^. CB loss is also a well-known feature of temporal lobe epilepsy^45–47^. Since neuronal hyperactivity is also associated with AD, it has been suggested that CB loss is a consequence of neuronal hyperactivity. However, the possibility that CB loss is not merely an epiphenomenon of these diseases but causally related to DG dysfunction remains to be elucidated. In this respect, CBKO mice can be a useful model for studying the pathophysiological significance of CB loss in various diseases. In this study, we proposed a causal link between Ca^2+^ buffering deficits and hyperexcitability of the DG, which leads to cognitive deficits. Furthermore, we identified the molecular mechanism by which Ca^2+^ buffering deficits lead to neuronal hyperexcitability. We showed that CB depletion or overexpression, respectively, in HT22 cells decreased or increased the level of surface Kv4.1 along with p-CaMKII levels (Fig. 5a, b). Furthermore, pharmacological inhibition of CaMKII reduced Kv4.1 surface expression (Fig. 5c), suggesting that Kv4.1 trafficking to the surface membrane is regulated by CaMKII-dependent phosphorylation. We further identified a phosphorylation site in Kv4.1 that was critical for channel trafficking (Fig. 7). However, it is unclear how reduced Ca^2+^ buffering inhibited CaMKII activity, since reduced Ca^2+^ buffering was expected to increase cytosolic Ca^2+^ levels, and CaMKII activity was expected to increase at high Ca^2+^. To investigate this, we focused on another Ca^2+^-dependent enzyme that can act in the opposite way to kinase, calcineurin, a Ca^2+^-dependent phosphatase, and confirmed that reduced CaMKII activity and reduced Kv4.1 surface expression were restored, at least in part, with the pharmacological inhibition of calcineurin (Fig. 5d). These results suggest that Ca^2+^ dysregulation caused by CBKO resulted in an abnormal balance between Ca^2+^-dependent protein phosphatase and Ca^2+^-dependent protein kinase, causing a decrease in Kv4.1 phosphorylation, which is crucial for the surface localization of Kv4.1.

Protein phosphorylation and dephosphorylation are two essential mechanisms that regulate many functional proteins, such as enzymes, receptors, and ion channels. In the brain, Ca^2+^-dependent kinases and phosphatases play key roles in the activity-dependent regulation of neuronal functions. In particular, their roles in synaptic plasticity are well-known. A small increase in Ca^2+^ activates phosphatases, leading to long-term depression, while a higher increase in Ca^2+^ activates kinases, leading to long-term potentiation^48^. These results imply that Ca^2+^ signals are translated into the balance between Ca^2+^-dependent kinases and phosphatases, thus determining the phosphorylation states of synaptic proteins to regulate synaptic plasticity. Activity-dependent regulation of ion channels through the balancing of Ca^2+^-dependent kinases and phosphatases has also been well documented. Activity-dependent downregulation of Kv1.2 activity in CA3 pyramidal neurons is mediated by Ca^2+^- and Src family kinase-dependent endocytosis, which is facilitated by Zn^2+^-mediated suppression of tyrosine phosphatase^49,50^. The surface expression of Kv4.2 and endogenous A-type K^+^ currents in hippocampal neurons was increased by the introduction of constitutively active CaMKII^51^. Taken together, these and our results suggest that the phosphorylation-dependent surface localization of Kv channels, rather than their expression, might be an important regulatory mechanism modulating neuronal activity. However, we found that the levels of surface Kv4.2 proteins were not altered by the CB levels (Fig. 5a, b). Further studies are needed to clarify the difference between CaMKII-dependent regulation of Kv4.1 and Kv4.2. It is possible that the increase in CaMKII activity due to CB expression is not sufficient to increase Kv4.2 phosphorylation to promote surface trafficking. We performed experiments without applying specific stimuli that are usually used in synaptic plasticity experiments to promote Ca^2+^ influx, while previous studies mainly focused on the role of Ca^2+^-dependent kinases and phosphatases in activity-dependent regulation. Therefore, our results represent the impact of the balance between Ca^2+^-dependent kinases and phosphatases on the phosphorylation states of Kv4.1 at resting Ca^2+^ levels. It remains to be investigated how the Kv4.1 phosphorylation state is changed upon stimulation, contributing to the activity-dependent regulation of GC excitability.

It has been established that CaMKII is dysregulated in the hippocampus of patients with AD^52^. Key findings include reduced autophosphorylation of CaMKII in the hippocampus and frontal cortex of patients with AD^53^, alterations in the subcellular localization of autophosphorylated CaMKII in the CA3 and DG of the AD-affected brain^18^, and inhibition of CaMKII activation by amyloid β^54^. However, it is still unclear how this dysregulation occurs. In a recent study, we showed that Tg2576 mice exhibited impaired pattern separation and hyperexcitable DG at the early preclinical stage^39^. These findings are comparable to those observed in CBKO mice in the present study. The similar phenotypes of Tg2576 and CBKO mice suggest that they share a common mechanism, and we found that Ca^2+^ buffering capacity is severely reduced in GCs from Tg2576 mice (Fig. 8). Considering that CaMKII activation is inhibited by CB depletion (Fig. 5), reduced Ca^2+^ buffering capacity in Tg2576 GCs may contribute, at least in part, to dysregulated CaMKII signaling in AD. It is interesting to note that mice lacking CaMKII or calcineurin exhibit multiple abnormal behaviors related to schizophrenia^11,55^. These results together with the present study support the view that Ca^2+^ buffering deficits are causally related to hyperexcitable DG and cognitive deficits. However, abnormal excitability and CB depletion in the DG were also reported in CaMKII-deficient mice^11^, suggesting the possibility that CB expression is regulated by CaMKII. It is possible that CaMKII activity is regulated by Ca^2+^ and that activated CaMKII, in turn, regulates various mechanisms that regulate Ca^2+^ homeostasis, including CB expression. Close interrelationships between Ca^2+^ homeostasis and the balance between excitation and inhibition may be the most important features underlying normal brain functions. Considering that numerous Ca^2+^ buffers are present in neurons, future studies should examine the role of different Ca^2+^ buffers in this relationship.

## References

[1] Schwaller B (2012). The use of transgenic mouse models to reveal the functions of Ca2+ buffer proteins in excitable cells. Biochimica et. Biophysica Acta (BBA)-Gen. Subj..

[2] Celio M (1990). Calbindin D-28k and parvalbumin in the rat nervous system. Neuroscience.

[3] Lee SH, Ho W-K, Lee S-H (2009). Characterization of somatic Ca2+ clearance mechanisms in young and mature hippocampal granule cells. Cell Calcium.

[4] Blatow M, Caputi A, Burnashev N, Monyer H, Rozov A (2003). Ca2+ buffer saturation underlies paired pulse facilitation in calbindin-D28k-containing terminals. Neuron.

[5] Iacopino A, Christakos S, Modi P, Altar C (1992). Nerve growth factor increases calcium binding protein (calbindin-D28K) in rat olfactory bulb. Brain Res..

[6] Iacopino A, Christakos S, German D, Sonsalla P, Altar C (1992). Calbindin-D28K-containing neurons in animal models of neurodegeneration: possible protection from excitotoxicity. Mol. brain Res..

[7] Iacopino AM, Christakos S (1990). Specific reduction of calcium-binding protein (28-kilodalton calbindin-D) gene expression in aging and neurodegenerative diseases. Proc. Natl Acad. Sci. USA.

[8] Palop JJ (2003). Neuronal depletion of calcium-dependent proteins in the dentate gyrus is tightly linked to Alzheimer’s disease-related cognitive deficits. Proc. Natl Acad. Sci. USA.

[9] Stefanits H, Ebetsberger-Dachs G, Weis S, Haberler C (2014). Medulloblastoma with multi-lineage differentiation including myogenic and melanotic elements: a case report with molecular data. Clin. Neuropathol..

[10] Altar CA (2005). Deficient hippocampal neuron expression of proteasome, ubiquitin, and mitochondrial genes in multiple schizophrenia cohorts. Biol. Psychiatry.

[11] Yamasaki N (2008). Alpha-CaMKII deficiency causes immature dentate gyrus, a novel candidate endophenotype of psychiatric disorders. Mol. Brain.

[12] Walton N (2012). Detection of an immature dentate gyrus feature in human schizophrenia/bipolar patients. Transl. Psychiatry.

[13] Li J-T (2017). Suppressed calbindin levels in hippocampal excitatory neurons mediate stress-induced memory loss. Cell Rep..

[14] Bayer KU, Schulman H (2019). CaM kinase: still inspiring at 40. Neuron.

[15] Hund TJ (2010). A β IV-spectrin/CaMKII signaling complex is essential for membrane excitability in mice. J. Clin. Investig..

[16] Zybura AS, Baucum AJ, Rush AM, Cummins TR, Hudmon A (2020). CaMKII enhances voltage-gated sodium channel Nav1. 6 activity and neuronal excitability. J. Biol. Chem..

[17] Küry S (2017). De novo mutations in protein kinase genes CAMK2A and CAMK2B cause intellectual disability. Am. J. Hum. Genet..

[18] Reese LC, Laezza F, Woltjer R, Taglialatela G (2011). Dysregulated phosphorylation of Ca2+/calmodulin‐dependent protein kinase II‐α in the hippocampus of subjects with mild cognitive impairment and Alzheimer’s disease. J. Neurochem..

[19] Winder DG, Sweatt JD (2001). Roles of serine/threonine phosphatases in hippocampal synaptic plasticity. Nat. Rev. Neurosci..

[20] Kim JH (2006). Overexpression of calbindin‐D28K in hippocampal progenitor cells increases neuronal differentiation and neurite outgrowth.. FASEB J..

[21] Leutgeb JK, Leutgeb S, Moser MB, Moser EI (2007). Pattern separation in the dentate gyrus and CA3 of the hippocampus. Science.

[22] O’Reilly RC, McClelland JL (1994). Hippocampal conjunctive encoding, storage, and recall: avoiding a trade-off. Hippocampus.

[23] Treves A, Rolls ET (1994). Computational analysis of the role of the hippocampus in memory. Hippocampus.

[24] Rolls ET (2013). The mechanisms for pattern completion and pattern separation in the hippocampus. Front. Syst. Neurosci..

[25] Kim K-R (2020). Kv4. 1, a key ion channel for low frequency firing of dentate granule cells, is crucial for pattern separation. J. Neurosci..

[26] Lee SH (2012). Impaired short-term plasticity in mossy fiber synapses caused by mitochondrial dysfunction of dentate granule cells is the earliest synaptic deficit in a mouse model of Alzheimer’s disease. J. Neurosci..

[27] Neher E (1998). Usefulness and limitations of linear approximations to the understanding of Ca++ signals. Cell Calcium.

[28] Neher E, Augustine G (1992). Calcium gradients and buffers in bovine chromaffin cells. J. Physiol..

[29] Cesarini E (2018). Melatonin protects hippocampal HT22 cells from the effects of serum deprivation specifically targeting mitochondria. PLoS ONE.

[30] Pyun J-H (2018). Cardiac specific PRMT1 ablation causes heart failure through CaMKII dysregulation. Nat. Commun..

[31] Jeong M-H (2019). PRMT1 suppresses ATF4-mediated endoplasmic reticulum response in cardiomyocytes. Cell Death Dis..

[32] Choi S (2019). Skeletal muscle-specific Prmt1 deletion causes muscle atrophy via deregulation of the PRMT6-FOXO3 axis. Autophagy.

[33] Bae, J. H. et al. Satellite cell‐specific ablation of Cdon impairs integrin activation, FGF signalling, and muscle regeneration. *J. Cachexia Sarcopenia Muscle* **11**, 1089–1103 (2020). 10.1002/jcsm.12563 PMC7432598 32103583

[34] McHugh, T. J., Jones, M. W., Quinn, J. J., Balthasar, N., Coppari, R., Elmquist, J. K., et al. Dentate gyrus NMDA receptors mediate rapid pattern separation in the hippocampal network. *Science*. **317**, 94–99 (2007). 10.1126/science.1140263 17556551

[35] McNaughton, B. L. & Nadel, L. Hebb-Marr networks and the neurobiological representation of action in space. *Neuroscience and connectionist theory*, 1–63 (1990).

[36] Kim S, Mátyás F, Lee S, Acsády L, Shin H-S (2012). Lateralization of observational fear learning at the cortical but not thalamic level in mice. Proc. Natl Acad. Sci. USA.

[37] Rolls ET, Kesner RP (2006). A computational theory of hippocampal function, and empirical tests of the theory. Prog. Neurobiol..

[38] Ohno M, Sametsky EA, Silva AJ, Disterhoft JF (2006). Differential effects of αCaMKII mutation on hippocampal learning and changes in intrinsic neuronal excitability. Eur. J. Neurosci..

[39] Kim K-R (2021). Impaired pattern separation in Tg2576 mice is associated with hyperexcitable dentate gyrus caused by Kv4. 1 downregulation. Mol. Brain.

[40] Cedervall T (2005). Redox sensitive cysteine residues in calbindin D28k are structurally and functionally important. Biochemistry.

[41] Molinari S (1996). Deficits in memory and hippocampal long-term potentiation in mice with reduced calbindin D28K expression. Proc. Natl Acad. Sci. USA.

[42] Yang, C. H., Lee, K. H., Ho, W. K. & Lee, S. H. Inter‐spike mitochondrial Ca2+ release enhances high frequency synaptic transmission. *J. Physiol.* **599**, 1567–1594 (2021). 10.1113/JP280351 33140422

[43] Airaksinen MS (1997). Ataxia and altered dendritic calcium signaling in mice carrying a targeted null mutation of the calbindin D28k gene. Proc. Natl Acad. Sci. USA.

[44] Dumas T, Powers E, Tarapore P, Sapolsky R (2004). Overexpression of calbindin D28k in dentate gyrus granule cells alters mossy fiber presynaptic function and impairs hippocampal‐dependent memory. Hippocampus.

[45] Carter DS, Harrison AJ, Falenski KW, Blair RE, DeLorenzo RJ (2008). Long-term decrease in calbindin-D28K expression in the hippocampus of epileptic rats following pilocarpine-induced status epilepticus. Epilepsy Res..

[46] Maglóczky Z, Halasz P, CZIRJÁK JVS, Freund T (1997). Loss of calbindin-D, immunoreactivity from dentate granule cells in human temporal lobe epilepsy. Neuroscience.

[47] Nägerl UV (2000). Surviving granule cells of the sclerotic human hippocampus have reduced Ca2+ influx because of a loss of calbindin-D28k in temporal lobe epilepsy. J. Neurosci..

[48] Lisman J (1989). A mechanism for the Hebb and the anti-Hebb processes underlying learning and memory. Proc. Natl Acad. Sci. USA.

[49] Eom K (2019). Intracellular Zn2+ signaling facilitates mossy fiber input-induced heterosynaptic potentiation of direct cortical inputs in hippocampal CA3 pyramidal cells. J. Neurosci..

[50] Hyun JH, Eom K, Lee KH, Ho WK, Lee SH (2013). Activity‐dependent downregulation of D‐type K+ channel subunit Kv1. 2 in rat hippocampal CA3 pyramidal neurons. J. Physiol..

[51] Varga AW (2004). Calcium–calmodulin-dependent kinase II modulates Kv4. 2 channel expression and upregulates neuronal A-type potassium currents. J. Neurosci..

[52] Ghosh A, Giese KP (2015). Calcium/calmodulin-dependent kinase II and Alzheimer’s disease. Mol. Brain.

[53] Amada N, Aihara K, Ravid R, Horie M (2005). Reduction of NR1 and phosphorylated Ca2+/calmodulin-dependent protein kinase II levels in Alzheimer’s disease. Neuroreport.

[54] Zhao D, Watson JB, Xie C-W (2004). Amyloid β prevents activation of calcium/calmodulin-dependent protein kinase II and AMPA receptor phosphorylation during hippocampal long-term potentiation. J. Neurophysiol..

[55] Miyakawa T (2003). Conditional calcineurin knockout mice exhibit multiple abnormal behaviors related to schizophrenia. Proc. Natl Acad. Sci. USA.

